# Effects of common rice field weeds on the survival, feeding rate and feeding behaviour of the crayfish *Procambarus clarkii*

**DOI:** 10.1038/s41598-021-98936-3

**Published:** 2021-09-29

**Authors:** Quan Yuan, Zhihui Tian, Weiwei Lv, Weiwei Huang, Xiaolin Sun, Weiguang Lv, Yonghong Bi, Guohui Shen, Wenzong Zhou

**Affiliations:** 1 Eco-environmental Protection Research Institute, Shanghai Academy of Agricultural Sciences, 1000 Jinqi Road, Shanghai, 201403 China; 2 State Key Laboratory of Fresh Water Ecology and Biotechnology, Institute of Hydrobiology, Chinese Academy of Sciences, Wuhan, 430072 China

**Keywords:** Animal behaviour, Ichthyology

## Abstract

To check if it is possible for crayfish to reduce the weed biomass in a paddy field, we hypothesised that crayfish can feed on common weeds in a paddy field. The feeding ability of red swamp crayfish, *Procambarus clarkii*, males and females for 4 weeds, *Ludwigia prostrata* Roxb., *Leptochloa chinensis* (L.) Nees, *Echinochloa crusgalli* (L.) Beauv and *Eclipta prostrata* L., commonly found in rice–crayfish fields were evaluated using a quantitative feeding experiment and behaviour observation experiment. The results of the quantitative feeding and behaviour experiments were highly consistent. The *P. clarkii* gender and weed species had no interactive effects on survival rate, the daily feed intake (FI) and percentage of daily feed intake (PFI). The results of the quantitative feeding experiment showed that the FI and PFI values of both *P. clarkii* females and males were significantly higher in the *P. clarkii* feed group than in the weed treatment group. Both FI and PFI were significantly higher in the *L. chinensis* group than in the other treatment groups. The survival rate of *P. clarkii* was significantly lower in the *E. crusgalli* group than in the other treatment groups. The behaviour observation experiment showed that the feeding frequency and duration were in the order of *L. chinensis* > *E. prostrata* > *L. prostrata* > *E. crusgalli*. The results indicate that the *P. clarkii* specimens liked to eat *L. chinensis* (mean PFI was more than 2%), hardly fed on *E. crusgalli*.

## Introduction

The red swamp crayfish, *Procambarus clarkii* (Girard, 1852), is an economically important species in China. In 2018, the output of *P. clarkii* in China was 1.64 million tonnes, so it ranked first in the output of freshwater crustaceans in China; in addition, the output was nearly double the annual output in 2016^1,2^. In 2018, the total area for *P. clarkii* cultivation in China was 1.12 million hectares, and, of this, paddy fields accounted for 0.84 million hectares. Paddy fields accounted for 40% of the total area used for rice cultivation and fisheries in 2018^3^. Rice–crayfish co-culture has a lot of economic benefits, with an increase in net income by 6302.7 USD per hectare^4^. According to our field survey (data not provided), farmers in some places such as Honghu City in Hubei Province and Yancheng City in Jiangsu Province often widen rice ditches and pay more attention to *P. clarkii* cultivation than rice production, which is against the principles of rice–fish co-culture (Technical specifications for integrated farming of rice and aquaculture animal, SC/T 1135-2017).

Since the twenty-first century, rice–fish co-culture has been widely practiced in paddy fields in Asian countries, especially in China^5^. The term ‘fish’ refers to a wide range of aquatic animals such as carp, crab and crayfish. Previous studies have suggested that aquaculture animals ushered into paddy fields can provide multiple services to rice ecosystems, i.e. decrease the abundance of insects and weeds, reduce agrochemicals inputs and enhance both soil and rice quality^5–8^. The aims of rice–fish co-culture are to ensure the stable yield of rice and achieve stable gain and income through the cultivation of aquatic animals. Therefore, it is important to study the ecological services provided by aquatic organisms, such as weed control, in a rice–crayfish co-culture system.

Weed damage has become an important factor that affects rice production, potentially reducing rice yield by 20–80%^9^. Chemical control is the main method used to control weeds^10^, however, the wide application of chemical herbicides has caused environmental problems such as pollution, drug resistance and residual toxicity^11–13^. This has created a demand for sustainable crop management. Previous field studies have shown that rice–crayfish farming has a good control effect on dominant weed species, such as *Ludwigia prostrata*, *Ammannia baccifera*, *Leptochloa chinensis*, *Lindernia procumbens* and *Echinochloa crusgalli*^14,15^. Xu et al.^15^ found that Dicotyledoneae and Gramineae weed densities after 2–3 years of integrated rice–crayfish farming decreased by 73.53% and 63.26%, respectively, when compared with the rice monoculture model. Nonetheless, there is still a lack of direct evidence for *P. clarkii* control of weeds. In this study, we hypothesized that crayfish can directly feed on weeds found in rice fields in China. We evaluated the ability of *P. clarkii* to feed on common weeds found in rice fields in China by direct feeding and observation of animal behaviour and determined the specific amount of different weeds fed on by *P. clarkii*.

## Materials and methods

### Animals and weeds

The experiments were conducted in the laboratory of the Eco-environmental Protection Research Institute, Shanghai Academy of Agricultural Sciences. The *P. clarkii* specimens used in this study were obtained from Zhuanghang Experimental Station of Shanghai Academy of Agricultural Sciences. About 500 *P. clarkii* (9.76 ± 4.42 g) specimens were transported to the laboratory and acclimated for 2 weeks in plastic boxes (46.4 × 34.7 × 22 cm, L × W × H) filled with tap water. In the first week of acclimatization, the *P. clarkii* specimens were fed excessively with pelleted commercial crayfish feed (38% crude protein) every day. In the second week of acclimatization, the *P. clarkii* specimens were fed excessively with weeds every 2 days for domestication. Animal welfare and experimental procedures were in accordance with the Guide for the Care and Use of Laboratory Animals [Ministry of Science and Technology of China, (2006)398].

The following plant species were selected on the basis of their status as common weeds found in local rice–crayfish farms^15^^,^^14^: *Ludwigia prostrata* Roxb., *Leptochloa chinensis* (L.) Nees, *Echinochloa crusgalli* (L.) Beauv and *Eclipta prostrata* L. Mature seeds of the 4 weed types were collected from a field located at the Zhuanghang Experimental Station of the Shanghai Academy of Agricultural Sciences. Botanist Dr Tian was responsible for the collection and identification of the weed seeds. The seeds were air-dried and peeled using physical percussion. After collection, the seeds were separated and purified by air separation. The purified seeds were stored in the refrigerator at 4 °C in the weed laboratory of the Eco-Environmental Protection Research Institute, Shanghai Academy of Agricultural Sciences. The voucher IDs of the 4 weeds are D201903, Q201903, B201903 and L201903. The weeds were planted in trays (length × width × height, 60 × 24 × 3 cm) 1 month before the experiment. The trays were filled with soil sterilized by dry heat and placed on a plastic chassis, distilled water was added to the soil in the trays till saturation, and the weed seeds were then evenly spread. The trays were placed in a plant culture room with a photoperiod of 14 h light: 10 h dark and relative humidity of 60%. During the cultivation, water evaporation was monitored, and water was supplemented regularly. The plant experiments were performed in accordance with relevant guidelines.

### Quantitative feeding experiment

A total of 30 plastic boxes and 120 *P. clarkii* specimens of the same size (Table 1) were used for the quantitative feeding experiment. On the basis of the density of *P. clarkii* in rice–crayfish fields, 4 *P. clarkii* (about 4 times the field density) were raised in a plastic box (38 × 26 × 12.8 cm, L × W × H). *P. clarkii* males and females were fed the 4 weeds separately; each group was fed with 1 weed, with a pelleted commercial crayfish feed (38% crude protein) as the control. The experiment had 10 treatments, with 3 replicates of each treatment.

**Table 1:** Summary statistics (mean ± SD) of feeding related parameters for *P. clarkia* after different treatments.

Treatment group		Initial body length (mm)	Initial body weight (g)	Survival (%)	FI (g/ind.)	PFI (%)
Crayfish feed	Female	63.93 ± 4.55	6.43 ± 1.31	100%^a^	0.20 ± 0.060^a^	2.95 ± 0.74^a^
	Male	62.57 ± 5.08	6.84 ± 2.21	100%^a^	0.22 ± 0.015^a^	3.28 ± 0.85^a^
*L. chinensis*	Female	70.04 ± 7.39	8.55 ± 1.90	91.67 ± 14.43^a^	0.17 ± 0.067^b^	2.01 ± 0.79^b^
	Male	68.03 ± 7.80	7.47 ± 2.41	83.33 ± 28.87^a^	0.18 ± 0.10^b^	2.45 ± 1.36^b^
*L. prostrata*	Female	61.62 ± 8.64	5.78 ± 2.24	83.33 ± 28.87^a^	0.085 ± 0.046^c^	1.48 ± 0.81^c^
	Male	66.20 ± 7.01	7.27 ± 2.58	91.67 ± 14.43^a^	0.13 ± 0.050^c^	1.72 ± 0.70^c^
*E. prostrata*	Female	67.08 ± 7.35	7.52 ± 2.77	91.67 ± 14.43^a^	0.098 ± 0.041^c^	1.30 ± 0.55^c^
	Male	65.02 ± 9.29	7.39 ± 2.94	91.67 ± 14.43^a^	0.081 ± 0.037^c^	1.09 ± 0.51^c^
*E. crusgalli*	Female	61.58 ± 8.15	6.18 ± 2.72	75.00 ± 0.00^b^	0.032 ± 0.015^d^	0.51 ± 0.24^d^
	Male	66.92 ± 9.09	8.24 ± 3.47	66.67 ± 14.43^b^	0.061 ± 0.034^d^	0.75 ± 0.42^d^

*FI* daily feed intake, *PFI* percentage of the daily feed intake to body weight.

Values with different superscripts in each column are significantly different (*P* < 0.05). FI (g/ind.) = [*W*_*i*_ − (*W*_*d*_/*W*_*c*_)]/*n*/*d*, where n is the total number of *P. clarkii*, and d is days between feedings. PFI (%) = *FI*/*W*, where *W* is the mean initial body weight of *P. clarkii* in a weed treatment group.

At the beginning of the experiment, the healthy *P. clarkii* specimens were weighed and randomly divided into plastic boxes. Each box contained 4 plastic plants (3 cm in diameter and 35 cm in length) separately fixed on a sinker as shelter. The boxes were filled with approximately 5 L of aerated tap water to a depth of 5 cm. The experiment lasted for 2 weeks. No water exchange occurred during the experiment. The pH was 8.02 ± 0.12; dissolved oxygen, 8.82 ± 0.17 mg/L; residual chlorine, < 0.05 mg/L and water temperature, 22.2 ± 0.96 °C. A photoperiod of 12 h light (8:30 to 20:30):12 h dark was maintained during the experiment, and light intensity at the water surface was 108–254 lx (light meter LM-332, Japan).

The weeds were fed to *P. clarkii* from 15:00 to 16:00. Before feeding, the weed weight (*W*_*i*_) was measured using an electronic balance. The weeds were offered to the specimens every 2 days, and the feeding amount was calculated according to the feeding conditions of *P. clarkia*; the feeding amount was 0.77 ± 0.12 g of each weed. Before each feeding, the remaining weeds were removed with a net, placed in a glass culture dish and dried in an oven at 60 °C for 48–72 h. The remaining weeds after drying (*W*_*d*_) were weighed. In addition, 1.0 g (fresh weight) of each weed was weighed and dried in the oven, and the dry weight of 1.0 g weed (*W*_*c*_) was calculated and used to measure the actual weed feed intake of *P. clarkii*. The *P. clarkii* commercial feed was fed to the specimens excessively at 16:00 every day. Before each feeding, the commercial feed was weighed with an electronic balance, and the feed quantity was recorded. At 9:00 every day, the remaining commercial feed was removed with a net, placed in a glass culture dish and dried in an oven at 60 °C for 48–72 h. In addition, 1.0 g of the commercial feed was weighed and placed in a glass culture dish, covered with water and maintained from 16:00 to 9:00, and then dried in an oven, and the dry weight of 1.0 g commercial feed was calculated and used to measure the actual commercial feed intake of *P. clarkii*. During the experiment, the dead *P. clarkii* and moulted shells were removed at 9:00 every day.

### Behaviour observation experiment

To avoid cannibalism among the crayfish, 1 *P. clarkii* (10.03 ± 0.87 g) was placed in a plastic box (38 × 26 × 12.8 cm, L × W × H), and water depth in the box with no shelter was 3 cm. Each group was fed with 1 weed (1.08 ± 0.11 g), with 8 treatments in the experiment and 8 replicates of each treatment. The behaviour of the crayfish in the plastic box was observed and analysed (ViewPoint Zebralab3.3, France). The feeding rhythm of *P. clarkii* occurs mainly at night and in the morning^16^^,^^17^, so the behaviour observation time was from 15:00 to 23:59 and from 00:00 to 10:00.

The behaviour data were analysed using videos (Stomer player, China). The *P. clarkii* specimens caught the weeds with their claws and first appendages: single feeding behaviour. The following parameters were recorded during the experiment: feeding frequency, time of feed intake and duration of *P. clarkii* feeding.

### Statistical analysis

The daily feed intake (FI) was calculated as follows:

$$ {\text{FI }}\left( {{\text{g}}/{\text{ind}}.} \right) \, = \, \left[ {W_{i} - \, \left( {W_{d} /W_{c} } \right)} \right]/n/d $$

where n is the total number of *P. clarkii*, and d is days between feedings.

The percentage of daily feed intake (PFI) was calculated as follows:

$$ {\text{PFI }}\left( \% \right) \, = { 1}00 \, *FI/W $$

where *W* is the mean initial body weight of *P. clarkii* in a weed treatment group.

The statistical analyses were performed using SPSS 23.0, and Origin 2017 (Origin Lab, USA) was used for plotting. FI and PFI were compared between weeds and *P. clarkii* gender by using two-way ANOVA, followed by the LSD multiple comparison test.

## Results

### Weed consumption

A summary of the feed intake experiment results is presented in Table 1. The survival rate of both *P. clarkii* females and males in the *P. clarkii* feed group was 100%, whereas *P. clarkii* specimens died in all the other treatment groups. The two-way ANOVA showed that the *P. clarkii* gender and weed species had no interactive effects on FI and PFI (*P* > 0.05; Table 2). No significant differences in FI and PFI were observed between the *P. clarkii* females and males (*P* > 0.05), but the FI and PFI values of different weed species were significantly different (*P* < 0.05; Table 2). The FI and PFI values of both *P. clarkii* females and males were significantly higher in the *P. clarkii* feed group than in the weed treatment groups (*P* < 0.05; Table 1). Both FI and PFI were significantly higher in the *L. chinensis* group than in the other weed treatment groups. No significant differences in FI and PFI values were observed between the *L. prostrata* and *E. prostrata* groups. The FI and PFI of the *P. clarkii* females and males were significantly lower in the *E. crusgalli* group than in the other groups (Table 1).

**Table 2:** Statistical analysis (two-way ANOVA) of the effects of gender and different weeds on the daily feed intake (FI) and proportion of daily feed intake (PFI) of *P. clarkii*.

Dependent variable	Effect	*df*	*F*	*P*
FI	Sex	1	2.711	0.102
	Weeds	4	39.414	**0.000**
	Sex versus weeds	4	0.864	0.488
PFI	Sex	1	1.846	0.177
	Weeds	4	46.276	**0.000**
	Sex versus weeds	4	0.599	0.664

*FI* daily feed intake, *PFI* percentage of the daily feed intake to body weight.

*Bold characters indicate statistical significance at α = 0.05.

### Feeding characteristics

**Figure 1:**
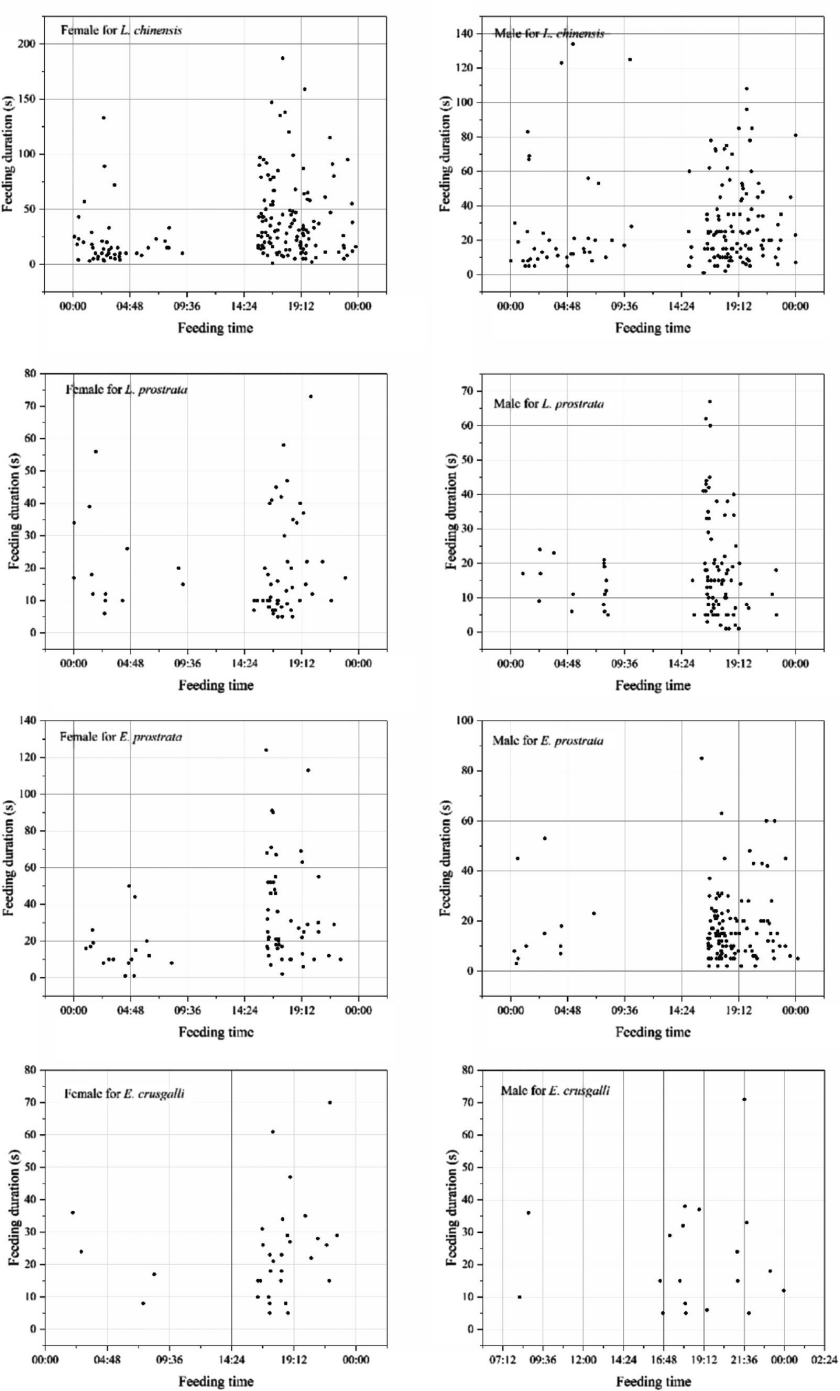
Feeding activity of *P. clarkii* during the observation period.

The commercial feed was basically consumed within 4 h after feeding in the feed intake experiment, so the control group was not used for the behaviour observation experiment. The behavioural analysis results are listed in Table 3. The feeding frequency and duration of *P. clarkii* on the different weed groups are as follows: *L. chinensis* > *E. prostrata* > *L. prostrata* > *E. crusgalli*. No significant differences in feeding frequency and feeding duration were observed between the *P. clarkii* females and males. Both *P. clarkii* females and males were more active from 17:00 to 20:00 (Fig. 1). Both feeding frequency and feeding duration were significantly higher in the *L. chinensis* group than in the *L. prostrata* and *E. crusgalli* groups (*P* < 0.05, Table 3). No significant differences in feeding frequency and feeding duration were observed between the *L. prostrata* and *E. crusgalli* groups (*P* > 0.05). The feeding activity of both *P. clarkii* females and males was the least in the *E. crusgalli* group (Table 3).

**Table 3:** Behavioural statistics of *P. clarkii* in different weed treatment groups (mean ± SD).

Weed treatment group	Repetition	Feeding frequency		Feeding duration (s)	
		Female	Male	Female	Male
*L. chinensis*	8	34.00 ± 24.40^a^	32.13 ± 20.86^a^	1228.75 ± 113.94^a^	935.88 ± 595.12^a^
*L. prostrata*	8	8.88 ± 4.30^b^	20.25 ± 20.70^ab^	175.00 ± 90.93^b^	349.13 ± 442.06^b^
*E. prostrata*	8	17.25 ± 10.31^ab^	25.88 ± 17.46^ab^	535.00 ± 523.25^ab^	443.88 ± 224.83^ab^
*E. crusgalli*	8	6.38 ± 4.93^b^	3.88 ± 2.59^b^	151.88 ± 108.76^b^	82.8 ± 55.49^b^

Values with different superscripts in each column are significantly different (*P* < 0.05).

The *P. clarkii* females and males of the *L. chinensis* group ate every hour during the observation period, and the feeding duration was longer from 19:00 to 20:00 and 4:00 to 5:00 for the males and from 18:00 to 19:00 and 2:00 to 4:00 for the females (Fig. 1). The *P. clarkii* males in the *L. prostrata* group fed frequently from 17:00 to 19:00, whereas the *P. clarkii* females fed frequently from 16:00 to 17:00. The *P. clarkii* males in the *E. prostrata* group fed frequently from 17:00 to 23:00, whereas the *P. clarkii* females fed frequently around 5:00. Both *P. clarkii* females and males in the *E. crusgalli* group showed the lowest feeding frequency and duration, but both *P. clarkii* females and males exhibited a relatively long feeding time from 21:00 to 22:00.

**Figure 2:**
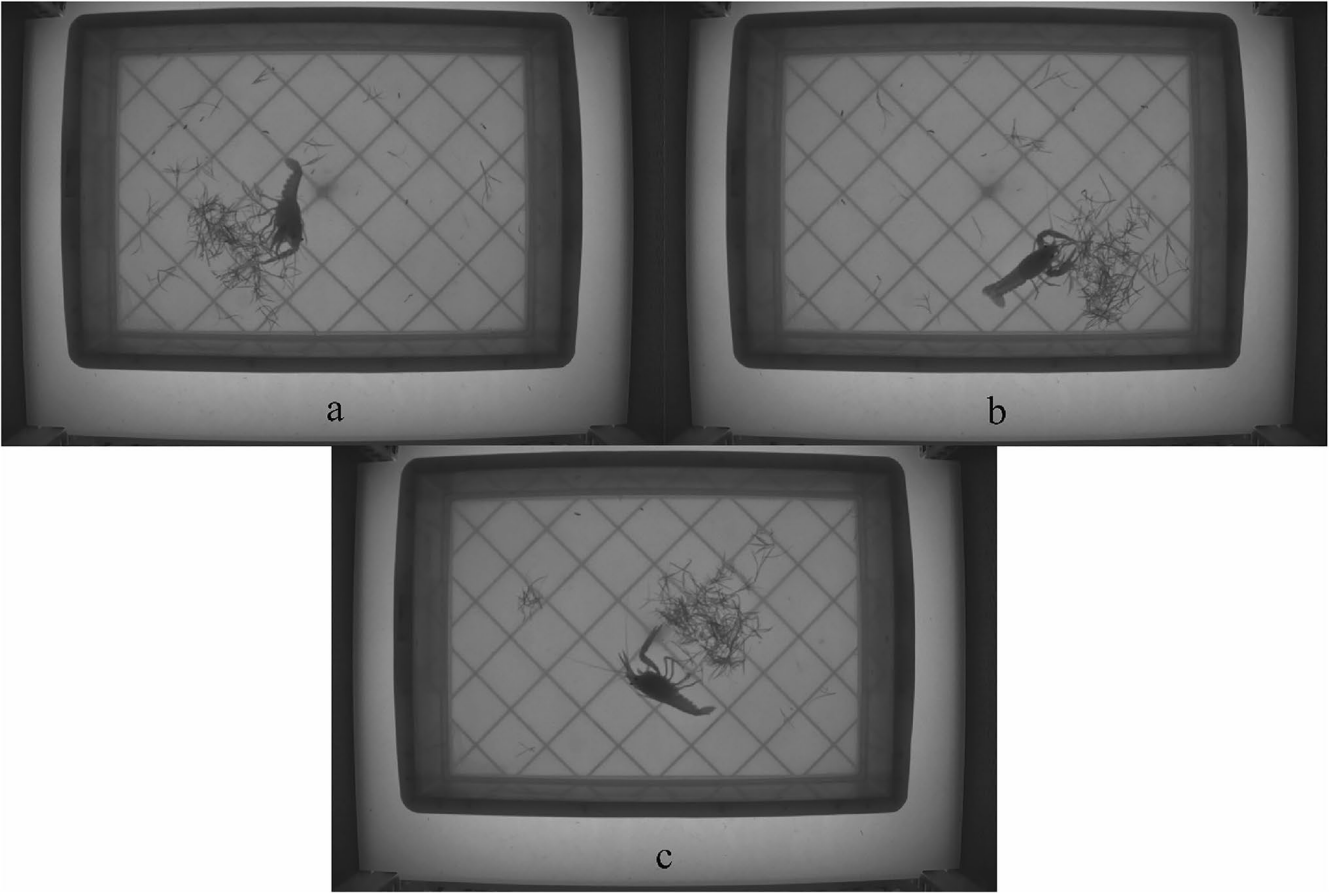
Feeding behaviour of *P. clarkii* (using *L. chinensis* as an example). (**a**) Feeding in the side direction, (**b**) Feeding in the front direction, (**c**) no motion.

*P. clarkii* exhibited 2 living states: active and static. When it started to move, it may have been looking for food. When it fed on the weeds, it used 2 positions: lying on its side for feeding (Fig. 2a) and feeding on its front (Fig. 2b). When *P. clarkii* fed on the weeds, it first used its cheliped to catch the weeds and then used its mouthparts to complete feeding (Fig. 2a, b). *P. clarkii* would lie on its side when it was still, for example, for sleeping (Fig. 2c).

## Discussion

*P. clarkii* is one of the most important invasive species found worldwide, and its ecological plasticity allows it to live in different types of environments. The great ecological plasticity of *P. clarkii* is also expressed in its feeding habits attributable to its polytrophic feeding behaviour^18^. Previous studies have shown that *P. clarkii* can establish a food chain suitable for its growth needs on the basis of the food sources in its living environment^19^. Therefore, is the fact that crayfish can reduce weed biomass in paddy fields related to this ecological characteristic? In this study, *P. clarkii* showed a strong appetite for some weeds, such as *L. chinensis*,and the PFI of *L. chinensis* was more than 2%. The results of the quantitative feeding and behaviour observation experiments were highly consistent. The *P. clarkii* specimens mostly preferred to eat *L. chinensis*, but hardly ate *E. crusgalli*. No significant differences were observed in the feeding amount with respect to *L. prostrata* and *E. prostrata*,however, according to the behaviour experiment results, the *P. clarkii* specimens preferred *E. prostrata*.

Previous studies on the diversity of weed communities in rice fields have shown that the density of *L. prostrata* and *E. prostrata* in the rice–crayfish co-culture system has significantly reduced when compared with rice monoculture, and the biomass of these 2 weeds continues to decrease with the increase in the duration of rice–crayfish co-culture^14,15,20^. In this study, the results showed that the feeding ability of *L. prostrata* and *E. prostrata* by *P. clarkii* was better and probably achieved by direct ingestion. However, the results obtained for *L. chinensis* and *E. crusgalli* were inconsistent with those of previous field studies. The biomass of *L. chinensis* and *E. crusgalli* decreased first (< 4 years) and then increased (7–8 years) with an increase in the duration of rice–crayfish co-culture, and both weed densities have been found to be lower in the rice–crayfish co-culture system than in the rice monoculture system^15,20^. This showed that *P. clarkii* can also control *L. chinensis* and *E. crusgalli*. However, in the present study, almost no *P. clarkii* specimen fed on *E. crusgalli*. In our study, increased feeding on *L. chinensis* was observed, relative to all the other weeds examined. Previous field investigations have reported that the biomass of *L. chinensis* was still significantly higher than that of *L. prostrata* and *E. prostrata*^15,20^, suggesting decreased consumption of *L. chinensis*. Therefore, it is unclear how *P. clarkii* controls weeds, especially *L. chinensis* and *E. crusgalli*, as it seems that the weeds were not controlled by direct feeding. *P. clarkii* can directly feed on agricultural seeds such as rice seeds, which contain high protein and/or energy^21,22^. Therefore, another possibility is that *P. clarkii* inhibits weed growth by ingesting weed seeds or suppressing weed seed germination through burrowing.

*P. clarkii* is an opportunistic, omnivorous feeder, and its diet includes submersed macrophytes, algae, invertebrates and detritus^18^. Generally, *P. clarkii* likes to feed on aquatic plants, but there as a few studies on *P. clarkii* feeding on terrestrial plants. During *P. clarkii* cultivation, fishermen generally grow aquatic plants such as *Hydrilla verticillata* and *Elodea nuttallii*. The large amount of *L. chinensis* consumed by *P. clarkii* in the present study shows the unlimited potential of *P. clarkii* to control weeds in rice fields. Both *L. chinensis* and *E. crusgalli* are gramineous plants, but the feeding selectivity of these 2 weeds with respect to *P. clarkii* was very different. *L. chinensis* seedlings are tender^23^, which may make its taste closer to that of aquatic plants. In addition, olfaction plays an important role in the feeding process of *P. clarkii*^24^, and it is possible that the odour of *L. chinensis* attracts *P. clarkii* to a greater extent than that of *E. crusgalli*. The results of the quantitative feeding experiment showed no significant differences in the feeding amount of the *P. clarkii* males and females in the *L. prostrata* and *E. prostrata* groups,however, the results of the behaviour observation experiment showed that the feeding frequency of the *P. clarkii* males was higher than that of the females in the *L. prostrata* and *E. prostrata* groups. *P. clarkii* males are more aggressive than the females^25^, so it is possible that the differences in their behaviour are because the *P. clarkii* males frequently move, search for food and eat.

All the weeds used in this study were newly grown seedlings. The appearance of weeds changes greatly in different growth stages. Therefore, the conclusions drawn on the basis of the *P. clarkii* specimens feeding on the weed seedlings in this study are not necessarily applicable to weeds in other growth stages. Freshwater crayfish have a dietary protein requirement of at least 30–35% for optimal growth^26^. The percentage of crude protein content in dry *L. chinensis* and *E. crusgalli* is 8.44% and 11.84%, respectively^27,28^. Although moulting of *P. clarkii* was observed in the weed groups in the present study, the *P. clarkii* specimens in the weed groups died with the extension of culture time, whereas the *P. clarkii* specimens in the feed group continued to grow well. Obviously, it is impossible for *P. clarkii* to feed on only weeds in a rice field, as the nutrition in weeds cannot completely meet the requirements for important life history events of *P. clarkii*, such as moulting and reproduction. However, the high ecological plasticity of *P. clarkii* makes its use in controlling weeds in rice fields possible. In a rice field, which has high amounts of plant material and low macroinvertebrate diversity, animal food is less important^18^. Therefore, the use of *P. clarkii* in controlling weeds in a rice field needs to be studied further.

## Conclusions

This study provides direct evidence that crayfish feed on common weeds in paddy fields, suggesting that crayfish may be used to reduce the weed biomass in paddy fields. *P. clarkii* preferred to eat *L. chinensis* and hardly fed on *E. crusgalli*. The mean percentage of daily feed intake to body weight per *P. clarkii* for *L. chinensis* was more than 2%.

## Supplementary Information

Supplementary Information.
