# Supplementary material for: Effects of common rice field weeds on the survival, feeding rate and feeding behaviour of the crayfish Procambarus clarkii
Source: Sci Rep. 2021 Sep 29;11:19327. doi: 10.1038/s41598-021-98936-3 (PMC8481556; doi:10.1038/s41598-021-98936-3)

Comparison of the beginning and end of *P. clarkii* feeding in the experimental observation period (A and B).


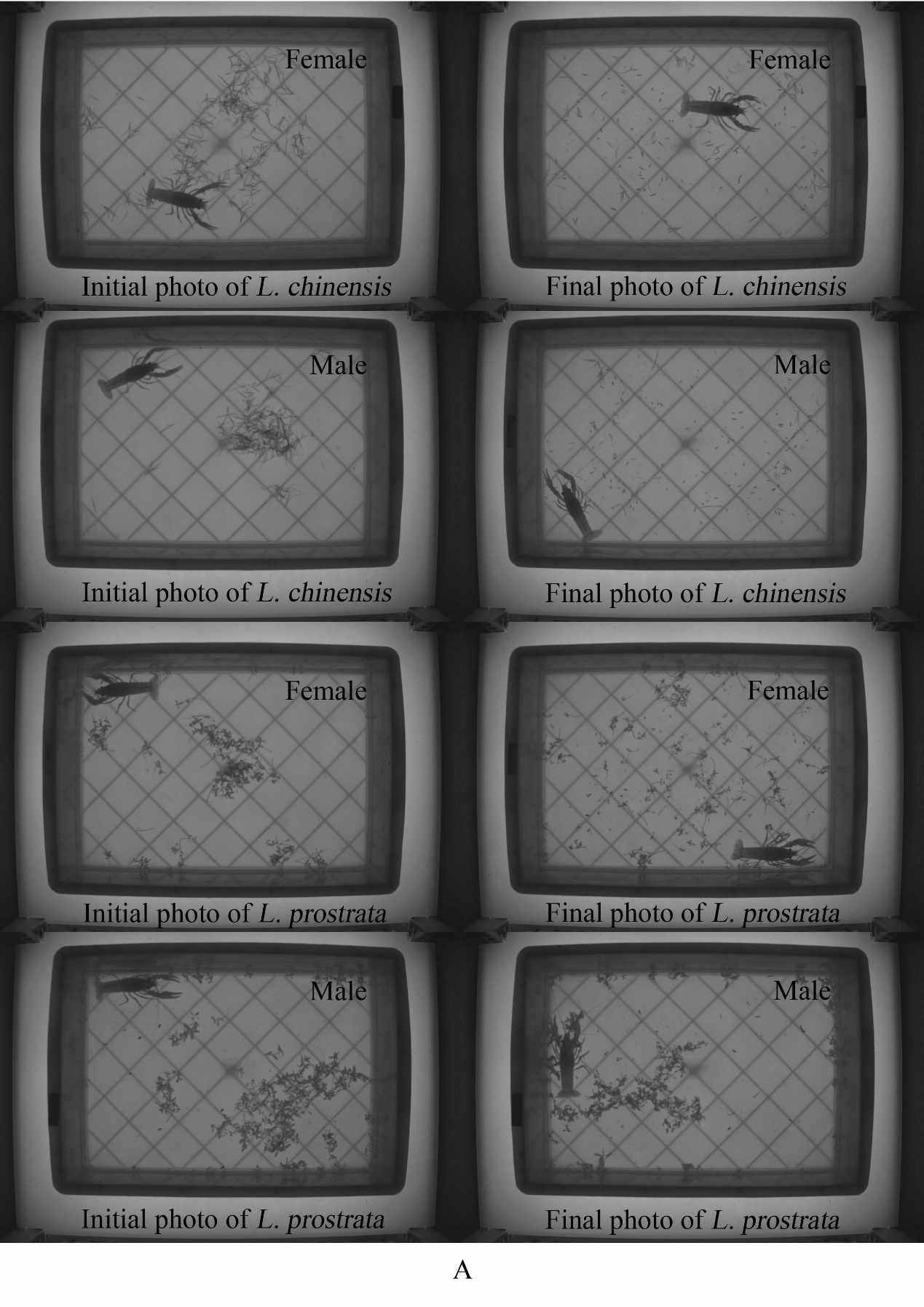


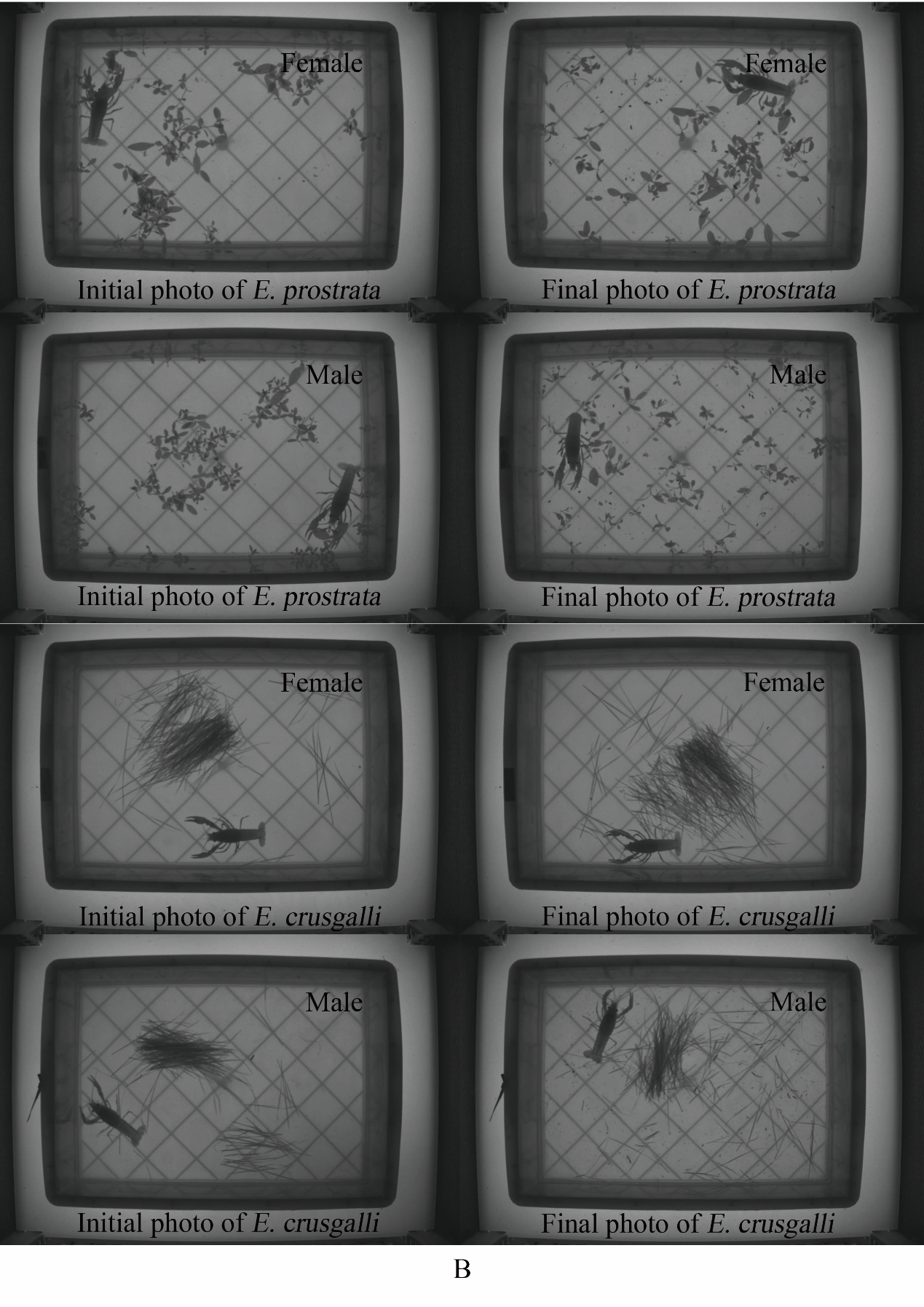

Supplement: Supplementary file 1 — Supplementary Information. [file 41598_2021_98936_MOESM1_ESM.docx]
